# Comparison of High-Charge Protocol vs. Dose Titration Protocol in Bilateral ECT: Evaluation of Antidepressant Effectiveness and EEG Parameters

**DOI:** 10.3390/jcm14186490

**Published:** 2025-09-15

**Authors:** Piotr Jażdżyk, Agnieszka Kuc, Albert Stachura, Agnieszka Segiet-Święcicka, Marcin Kosmalski, Łukasz Święcicki, Eric van Exel, Tadeusz Pietras

**Affiliations:** 1 Second Department of Psychiatry, Institute of Psychiatry and Neurology in Warsaw, 02-957 Warsaw, Poland; 2 Department of Psychiatry, Amsterdam UMC, Location Vrije Universiteit Amsterdam, 1081 HV Amsterdam, The Netherlands; 3 Department of Old Age Psychiatry, GGZinGeest Mental Health Care, 1062 HN Amsterdam, The Netherlands; 4 Center for Preclinical Research, Department of Methodology, Medical University of Warsaw, 02-097 Warsaw, Poland; 5 Department of Coronary Artery Disease and Cardiac Rehabilitation, Cardinal Stefan Wyszynski Institute of Cardiology, 04-628 Warsaw, Poland; 6 Department of Clinical Pharmacology, Medical University of Lodz, 90-419 Lodz, Poland; 7 Amsterdam Neuroscience, Mood, Anxiety, Psychosis, Stress and Sleep, 1081 HV Amsterdam, The Netherlands

**Keywords:** electroconvulsive therapy, high charge, dose titration, effectiveness, remission

## Abstract

**Objectives:** Recently, we modified the method of dosing charge in daily practice for patients undergoing bilateral electroconvulsive treatment (BL ECT). The aim of this study is to compare the effectiveness of two charges’ dosing protocols—High-Charge Protocol (HCP; based on the modified age-based method) and Dose Titration Protocol (DTP) in BL ECT for the treatment of patients with a depressive episode. **Methods:** The retrospective analysis compared the outcomes of BL ECT between patients receiving either HCP or DTP treatments. Patients’ mental status was assessed retrospectively using the Clinical Global Impression-Severity (CGI-S) and Clinical Global Impression-Improvement (CGI-I) scales. EEG parameters, including seizure duration and EEG ictal activity were analyzed. **Results:** When compared to DTP, the HCP group was older (55 years ± SD 15 vs. 41 years ± SD 17), had lower initial CGI-S (5 [IQR 5–6] vs. 6 [IQR 5–6]) and longer disease duration (15 years [IQR 7–20] vs. 9 years [IQR 3–18]). The DTP group had a higher percentage of remission (n = 17 [77.3%]) compared to the HCP group (n = 23 [43.4%]), with the same average number of sessions performed. In addition, the DTP group had significantly longer average seizure duration (68.6 s [IQR 52.7–84.7] vs. 38.4 s [IQR 33.8–47.1], adj. *p* < 0.001). **Conclusions:** Our results suggest that in BL ECT, administering high-charge protocols may have a detrimental impact on ECT effectiveness. Based on our findings, we propose adjusting the dosing in BL ECT according to the individual seizure threshold and avoiding frequent charge increases during the course of treatment.

## 1. Introduction

Electroconvulsive therapy (ECT), despite its overall effectiveness and safety, is often met with stigma and fear, mainly due to cognitive impairment and its invasive character. This strongly contributes to the limited use of ECT [1,2]. The effectiveness of ECT, as well as the presence and severity of its side effects, are likely related to the dosing of electric current in ECT sessions. Therefore, optimization of ECT dosage is required to improve the effectiveness of ECT and reduce the cognitive side effects. Although progress in the ECT technique has been made, dosing of the electric charge is still one of the most difficult aspects of performing ECT in daily clinical practice [3,4].

A recent analysis of patients who underwent either unilateral, bitemporal, or bifrontal ECT suggests that increasing the average electric charge during ECT sessions is associated with a higher remission rate [5]. This conclusion is supported by several studies in patients treated with the right unilateral (RUL) ECT technique [6,7,8]. McCall et al. showed a positive correlation between the stimulus dose and efficacy of ECT in reference to the experimentally measured seizure threshold (ST), which was defined as the minimal electrical stimulation needed to produce a general seizure of an adequate duration [6,9]. The authors concluded that the dependency in RUL ECT persisted with a 12-times multiplication of ST, leading to both higher response rates and increased cognitive side effects [6].

However, studies on the bilateral (BL) technique with the use of high charge are inconclusive. Sackeim et al. showed that ECT administered at a charge dose just above the ST was less effective in comparison to higher doses of 2.5 × ST after six ECT sessions; however, its overall effectiveness appeared to be equal at the end of the treatment [10]. Chanpattana et al. demonstrated that patients with schizophrenia treated with ECT exhibited equal response rates despite receiving different amounts of charge administered to the patient, namely 1 × ST, 2 × ST and 4 × ST [11]. However, the 4 × ST group had a lower number of days of ECT treatment. Therefore, the authors concluded that a higher multiplication of ST only accelerates the response to the treatment but does not affect the overall effectiveness [10,11].

Although the results of available studies may suggest that increasing the administered charge contributes to better clinical outcomes, it has not yet been verified how exceeding the seizure threshold by more than 2.5 times, and the related high charges, affects the effectiveness and remission rates in depressive patients treated with BL ECT. Moreover, based on the available data of RUL ECT from McCall (2000), there is the idea in daily clinical ECT practice that increasing energy in BL ECT, as in unilateral ECT, contributes to better treatment outcomes [6].

In January 2022, our department modified the dosing protocol for the BL ECT in daily clinical practice. This paradigm shift was implemented following an update to our internal ECT guidelines (according to van den Broek et al. [12]). The change of paradigm had also introduced to our clinical routine RUL as a treatment of first choice regarding ECT. From that point, BL electrode placement was reserved for patients who had an equal or higher than 6 Clinical Global Impression (CGI) Severity (CGI-S) baseline score or had BL ECT in the past. Therefore, we had the unique opportunity to test whether treatment with a high electric charge during BL ECT session would contribute to an increase or decrease in remission rates. We were able to do this by comparing the use of a high charge according to our previous protocol based on adjusting the dose of the energy to the age of the patient on the first session and further increasing the dosing on a regular basis (High-Charge Protocol; HCP), versus the typical Dose Titration Protocol (DTP) in which charge was based on individual titrated ST. As a result of this modification, we can assess the impact of differences in the average and cumulative charge doses on the rates of remission. This allows us to test the hypothesis that exceeding the therapeutic window in charge delivered during BL ECT sessions worsens treatment outcome, and may have a negative impact on seizure duration and EEG-based ictal activity.

## 2. Materials and Methods

### 2.1. Study Design

We retrospectively analyzed all consecutive patients who underwent BL ECT between January 2021 and January 2023 in the Institute of Psychiatry and Neurology, Warsaw, Poland. We included patients diagnosed with depression according to the Polish version of the International Classification for Diseases (ICD)-10 codes F32.1–F32.3, F33.1–F33.3, and F31.3–F31.5. Antidepressants and mood stabilizers were discontinued during the two-week qualification procedure prior to the start of treatments. Use of benzodiazepines was not allowed throughout the period of treatments. In cases of anxiety in patients before or during the ECT treatment, behavioral interventions or hydroxyzine was used. Only patients treated with BL ECT from the beginning of the treatment were included in the study population. ECT was administered twice a week by using a bidirectional constant current brief pulse (0.5–1.0 ms) Thymatron IV device (Somatics Inc. Lake Bluff, IL, USA). Two Thymatrons’ original programs were used during treatments, namely Double Dose (HCP) and DGX (DTP). In both programs, the increase in charge is reached by increasing the stimulus duration at first, and then by increasing the frequency of pulses in 10 Hz steps. During the procedure, the patients were premedicated with atropine 0.5 mg IV. The anesthetic differed between the groups, as described below. In all patients, succinylcholine (0.5–1.0 mg/kg) was chosen as a muscle relaxant. Patients were divided into two groups according to the protocol used in charge dosing. Informed consent for the use of anonymized clinical data for retrospective analysis was obtained from patients at the time of admission. The study complies with the 1964 Helsinki Declaration and its later amendments. Additionally, the study was approved by the Local Ethics Committee at the Institute of Psychiatry and Neurology (approval no. 29/2023).

### 2.2. High-Charge Protocol

In general, in the HCP, thiopental was used as the anesthetic agent, but at the discretion of the anesthesiologist, etomidate or propofol were also used. The preset program on which the treatment sessions were performed was the Thymatron Double Dose program, in which the pulse width varied from 0.5 to 1.0 ms. The amount of electric charge administered to the patient during the first ECT treatment corresponded to half of the age of the patient [13]. The energy doses were raised in consecutive treatments by adding 20% of the maximal output (108 mC) to the electrical charge used in the last session. Only if during the previous treatment session the seizure duration exceeded 60 s, the charge dosage stayed unchanged. The justification for this protocol in daily clinical practice was the fact that ST increases with successive treatments, and regularly increasing the charge was supposed to guarantee the highest possible treatment effectiveness (appropriately above the ST) [14]. This approach resulted in a lack of aborted (seizure duration < 25 s) or missed seizures; however, it almost inevitably achieved 200% (maximal output) in every patient after 10 to 12 sessions. Without the possibility of further increasing the charge dose, the procedures were continued until the patient achieved remission, further improvement was unlikely to appear, or the duration of the seizure was shorter than 15 s. The duration of the seizures was assessed based on a two-channel electroencephalogram (EEG).

### 2.3. Dose Titration Protocol

In the DTP group, etomidate (0.2–0.3 mg/kg) was used as the default anesthetic agent to counterbalance potential ST increase when the pulse width was preset to 1.0 ms. To find the individual ST of the patient, the dose titration procedure was performed at the first session. The typical titration protocol, which consisted of the 3 following steps of stimulations (50.4 mC, 100.8 mC, 151.2 mC), was applied in the first session. Lack of seizure activity in 30 s after the first stimulation was followed by a restimulation with charge at the next step. At least 15 s of ictal activity was considered an individual ST. In the consecutive sessions, the charge dose was computed by exceeding previously obtained STs by 2.5 times, and staying unchanged [12]. Only in cases of inadequate (<25 s of ictal activity) or missed seizures, the restimulation procedure was performed by increasing the charge by 50.4 mC or doubling the previously used dose, respectively.

### 2.4. Clinical Outcome

The clinical outcome of ECT sessions was determined using the CGI scale. It represents the physician’s overall judgment regarding patients’ status for specific diseases. This assessment is composed of 2 items: severity (CGI-S), which assesses the severity of symptoms, and improvement (CGI-I), which evaluates the symptom improvement after the treatments. The CGI-S and CGI-I scores were obtained retrospectively, based on the available medical records (daily notes and discharge letters) [15]. In assessing the CGI-S scores, two time points were considered, i.e., before the first session of ECT (CGI-S baseline), and immediately after the last session (CGI-S endpoint). Remission was defined as equal or less than 2 in the CGI-S [16,17,18]. Analysis of the ECT parameters included the total number of sessions, charge delivered, EEG-based seizure length, postictal suppression index (PSI), and midictal amplitude (MIA).

### 2.5. Statistical Analysis

The statistical analysis was performed with the SPSS software package (IBM SPSS Statistics 28.0.1.0, Chicago, IL, USA). Continuous variables are expressed as mean ± SD for normally distributed data or median with interquartile range [IQR] for non-normally distributed variables. The normality of the variables was assessed by examining the distributions and Q-Q plots. A *p*-value < 0.05 was considered statistically significant. For non-normally distributed measures, unadjusted between-group differences were summarized using the appropriate bivariate tests (Mann–Whitney U). To account for potential confounding, adjusted between-group comparisons were then estimated using multivariable ANCOVA, modeling CGI-S as approximately continuous and adjusting for treatment protocol, age, illness duration, baseline CGI-S, psychotic features, history of ECT, and psychiatric comorbidities (anxiety, substance use, personality disorder). CGI-I was analyzed within the same ANCOVA framework. As a sensitivity analysis, ΔCGI (baseline–endpoint) was modeled with the same covariates except baseline CGI-S, which was omitted to avoid mathematical coupling and reduce regression-to-the-mean bias.

Backward likelihood-ratio logistic regression served as the primary analytic model. Remission status (remitter vs. non-remitter) was the dependent variable, and the initial predictor set included type of protocol, disease duration, age, psychotic features, psychiatric comorbidities, history of ECT, and baseline CGI-S. Variables were removed stepwise (removal criterion *p*_out = 0.10) to obtain the final model. Finally, a dose-response curve was constructed by fitting a univariable logistic regression of remission on average dose per session and plotting the predicted probability curve. Observations were also stratified into quartiles of average dose; quartile-specific unadjusted remission proportions were plotted as points.

For the EEG secondary endpoints, each key parameter was analyzed using ANCOVA adjusting for the same baseline covariates (age, disease duration, baseline CGI-S, psychotic features, history of ECT, and psychiatric comorbidities), and multiple comparisons were controlled using the Benjamini–Hochberg FDR.

## 3. Results

### 3.1. Baseline Characteristics

**Figure 1:**
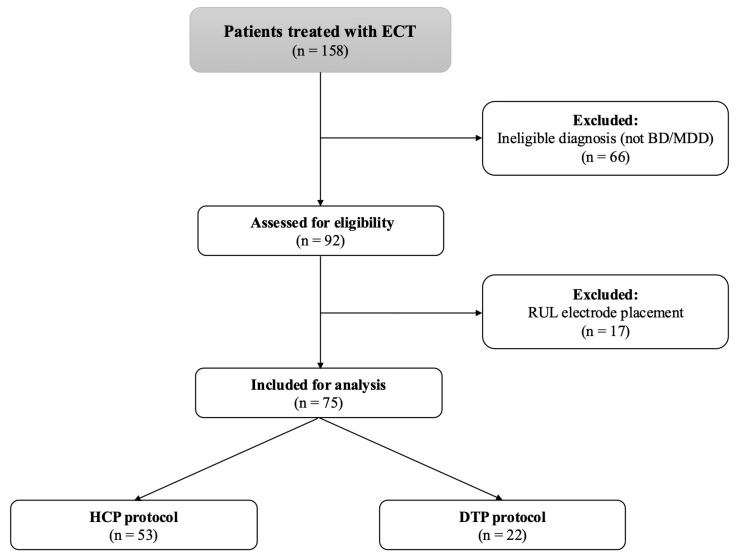
Study cohort flow diagram. BD—bipolar disorder; DTP—Dose Titration Protocol; ECT—electroconvulsive therapy; HCP—High-Charge Protocol; MDD—major depressive disorder; RUL—right unilateral electrode placement.

Figure 1 presents the flowchart of the study sample. Among 158 patients treated with ECT, 66 were excluded due to ineligible diagnoses (non-bipolar disorder; BD/major depressive disorder; MDD), leaving 92 patients assessed for eligibility. Of these, 17 were excluded because of right unilateral (RUL) electrode placement. The final study cohort comprised 75 patients, with 53 receiving the HCP protocol and 22 the DTP protocol (Figure 1). About half of the patients were diagnosed with bipolar disorder. An amount of 65% of patients were females, and the mean age was 51 ± 17 years. Detailed demographic characteristics are presented in Table 1.

The HCP and DTP groups did not differ in the distribution of gender (χ^2^ = 0.75; df = 2; *p* = 0.28), diagnosis of major depressive disorder (MDD) (χ^2^ = 0.006; df = 1; *p* = 0.57), psychotic features (χ^2^ = 2.59; df = 1; *p* = 0.09), and median index episode (4.5 [IQR 2–11.8] vs. 8 [IQR 3–24]; U = 426.5; *p* = 0.27). Patients in the DTP group were younger (41 ± 17 vs. 55 ± 15; t = 3.354; df = 73; *p* = 0.001), had a shorter median duration of disease (15 [IQR 7–20] vs. 9 [IQR 3–18]; t = 2.92; U = 320.5; *p* = 0.04), and higher median baseline CGI-S score (6 [IQR 5–6] vs. 5 [IQR 5–6]; U = 814.0; *p* = 0.004) when compared to the HCP group.

### 3.2. Effectiveness of ECT

**Figure 2:**
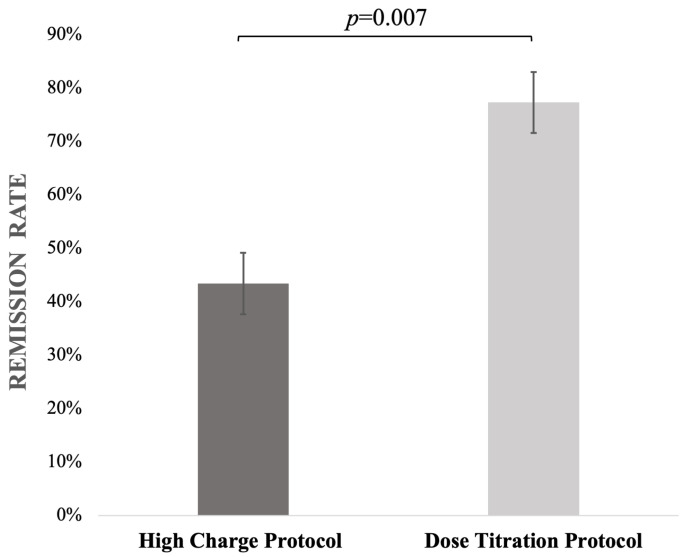
Remission rates in the High-Charge Protocol and Dose Titration Protocol groups.

Comparison between the HCP and DTP groups showed that the median CGI-S endpoint score (3 [IQR 2–3] vs. 2 [IQR 1.2–2.3]) and median of the CGI-I (2 [IQR 1–2] vs. 1 [IQR 1–2]) score were significantly higher in the HCP group (U = 344.0; *p* = 0.003; adj. *p* = 0.02 and U = 369.0; *p* = 0.007, adj. *p* = 0.02, respectively; Table 2). As a sensitivity analysis the mean change in CGI-S (∆CGI-S) was greater in the DTP than in the HCP group (3.96 ± 1.17 vs. 2.57 ± 1.29; t(73) = −4.34; *p* < 0.004; adjusted *p* < 0.001). The overall remission rates were 77.3% for the former and 43.4% for the latter group (χ^2^ = 7.169; df = 1; *p* = 0.007; Figure 2). Logistic regression analysis assessing the impact of potential confounding factors revealed that only dosing protocol was a significant predictor of remission status (β = 1.76 [1.06; 2.46]; *p* = 0.01; OR = 5.81 [1.48; 22.83]). In backward likelihood-ratio modeling, comorbid anxiety also remained in the model (β = –1.78; OR = 0.17; 95% CI 0.02–1.17; *p* = 0.07), although it did not reach statistical significance (Table 3). The full model including all initial predictors is provided in Supplementary Table S1.

**Figure 3:**
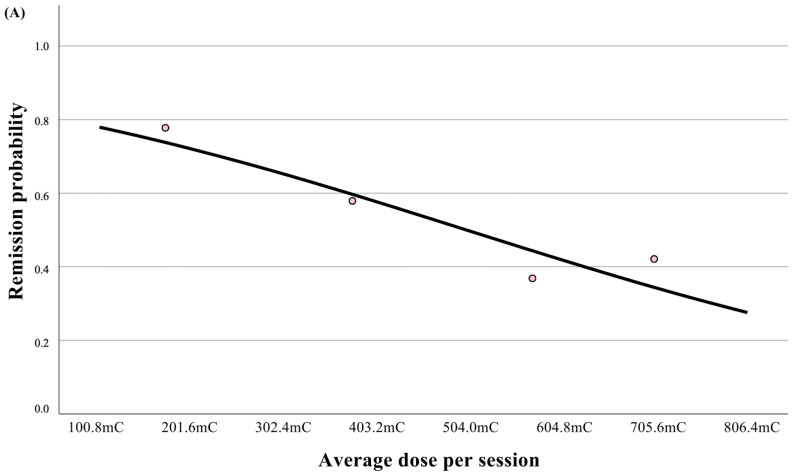

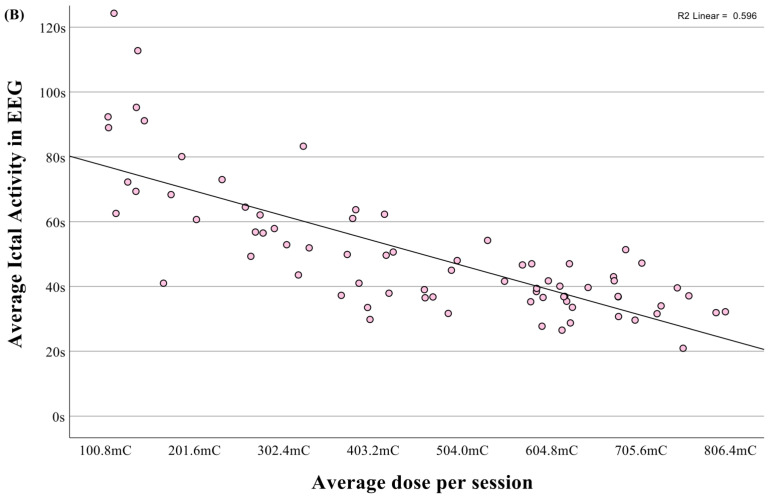
(**A**) Dose-response curve for remission versus average dose per session. The solid line is the fitted univariable logistic regression; points indicate quartile-specific unadjusted remission proportions (equal-frequency bins); (**B**) Scatterplot relating average (and cumulative) charge to mean seizure duration, with fitted linear regression lines to illustrate the trend. These displays are descriptive; formal inference derives from the prespecified ANCOVA (seizure duration) and logistic models (remission).

The dose-response curve showed a monotonic decrease in remission probability with an increasing average dose. Quartile points aligned with this trend, and no clear threshold was observed (Figure 3A).

### 3.3. Characteristics of Two Different Dosing Protocols

The cumulative charge administered to the patients was significantly higher in the HCP group when compared to that in the DTP group (5663.1 vs. 2088.4 mC, respectively; U = 134.0; *p* < 0.001; adj. *p* = 0.005; Table 4). While the median number of sessions performed was not significantly different between the groups (10.3 vs. 11 for HCP and DTP, respectively; t = −1.224; df = 73; *p* = 0.23; adj. *p* = 0.98), the average charge per session was almost three times higher in the HCP compared to that in the DTP (588.9 vs. 196.6 mC, respectively; U = 69.0; *p* < 0.001; adj. *p* < 0.001). In fact, in the HCP group, an average charge of 504 mC was achieved as fast as the fifth session, whereas in the DTP group, such a high charge was not achieved even during the whole course of the ECT treatment. Moreover, the HCP group had significantly different EEG parameters when compared to the DTP group.

To reduce the impact of repeated seizures, we analyzed separately the cumulative EEG parameters and average EEG parameter per session. In the DTP group, the average ictal duration was significantly longer (68.6 vs. 38.4 s in the HCP group; U = 1053.0; *p* < 0.001; adj. *p* < 0.001). The observed cumulative seizure duration throughout the completed course of ECT was significantly longer in the DTP group in comparison to that in the HCP group (675.5 vs. 399 s, respectively; U = 1039.5; *p* < 0.001; adj. *p* < 0.001). In the ANCOVA analysis of models for seizure metrics, dosing protocol was the only variable that remained statistically significant for both total seizure duration and average seizure duration, after adjustment for age, baseline CGI-S, disease duration, psychotic features, history of ECT, and psychiatric comorbidities (anxiety, substance use, personality disorder). No other covariate showed a significant association. To address multiplicity, *p*-values were adjusted using the Benjamini–Hochberg procedure to control the false discovery rate. Consistently, the exploratory dose-response curves in Figure 3B, based on average ictal EEG activity, demonstrated a parallel pattern, further supporting the association between stimulation parameters and seizure quality.

In the model including average PSI, the dosing protocol remained an independent predictor, with age also emerging as a significant predictor of PSI (F(1,67) = 5.9, *p* = 0.018, ηp^2^ = 0.08). Moreover, the average PSI was higher in the HCP group compared with that in the DTP group (74.2 vs. 63.6, respectively; U = 358.5; *p* = 0.02; adj. *p* = 0.008). By contrast, for total PSI, dosing protocol was not a significant predictor in either the unadjusted or adjusted analyses. Total MIA and average MIA showed numerically lower values in the HCP group compared with that in the DTP group (2108.9 vs. 1699.5, and 208.8 vs. 183.0, respectively), but these differences were not significant after adjustment (U = 773.0; *p* = 0.03; adj. *p* = 0.88 and U = 737.0; *p* = 0.07; adj. *p* = 0.85, respectively; Table 4).

## 4. Discussion

Our results indicate that using DTP in BL ECT leads to higher remission rates compared to the HCP strategy, despite a similar number of treatments and a significantly lower cumulative charge. Interestingly, this effect was observed even though the DTP group was significantly younger, which is contrary to prior findings suggesting ECT is more effective with increasing age [5].

To our knowledge, the only study which assessed the use of high absolute charges in BL ECT was a study by Ju et al. (Supplementary Table S2) [19]. The authors demonstrated that the use of charges exceeding 504 mC in patients with exceptionally high ST yielded a comparable ECT effectiveness to that of the group of patients for whom such high doses were not required. Although a trend towards higher efficacy of high charge approach was noted, the difference between the groups did not achieve statistical significance [19]. However, their methodology differed substantially from ours. In Ju et al.’s study, high charges were used only in patients with exceptionally high ST, and the dosing strategy applied just a 1.5× multiplication of ST. Furthermore, the high-dose group in that study received significantly more ECT sessions than the low-dose group (16.4 vs. 10.4), whereas session counts were consistent between groups in our study. These discrepancies limit the comparability of our findings, especially considering that Sackeim et al. (2020) demonstrated a positive correlation between the number of ECT sessions and increased response and remission rates [8].

Similarly, other studies examining different ST multiplication factors in the BL technique—such as those by Ottosson et al. and Chanpattana et al.—also found no significant differences in remission rates across groups. However, both studies suggested that higher suprathreshold dosing may lead to a faster clinical response [11,20]. In Ottosson’s study, patients were divided into three groups: just-above-threshold BL ECT, grossly suprathreshold BL ECT, and suprathreshold BL ECT with lidocaine pretreatment. While there were some indications that the grossly suprathreshold group experienced a faster clinical response, the authors concluded that both suprathreshold and grossly suprathreshold strategies were equivalent in terms of ECT efficacy [20]. Similarly, Chanpattana et al.compared groups receiving ECT at 1 ×, 2 ×, and 4 × ST, and found that while higher dosing led to a faster response—as reflected in the fewer sessions required—there were no significant differences in remission or response rates when assessed with the Brief Psychiatric Rating Scale (BPRS) [11]. Sackeim et al. (1993) likewise reported comparable remission outcomes between low- and high-charge BL ECT, using a high-dose group treated at 2.5 × ST [10]. Notably, in their study, charge manipulation was primarily achieved by increasing the frequency of pulses rather than adjusting the duration of the stimulation train. In contrast, our study prioritized modification of stimulus duration as the primary method of dose adjustment. This distinction enhances the clinical relevance of our findings, as modern ECT devices typically adjust charge by altering duration before modifying frequency.

Studies which focused on RUL ECT have shown mixed results. McCall et al. (2000) highlighted a positive correlation between the efficacy of RUL ECT and the degree to which the charge dose surpasses the ST [6]. On the other hand, Quante et al. (2011) assessed the effectiveness of various dosages (4 × ST, 7 × ST, and 10 × ST) in ultra-brief pulse RUL ECT and found no significant difference in improving depressive symptoms, although a noticeable trend was observed [21]. Importantly, in both studies, the average charges did not exceed 504 mC. Krystal et al. even showed a reduction in therapeutic response rates in the group of patients with the highest ST who needed a maximal possible charge dose (32% vs. overall 66%). However, multiplication of ST was equal to 2.25, so this should be considered a low-charge unilateral ECT, and at least part of the group did not elicit adequate seizures; therefore, the results are not fully comparable to our findings [22]. Results from Brus et al. suggested that patients with the highest average administrated charge also had the highest remission rates. However, these findings are also based on data extracted from patients without distinguishing between electrode placement (RUL or BL); no information about ST multiplication was provided, and the vast majority (90%) of the ECT sessions were performed using the RUL electrode placement technique [5].

While some studies examining the impact of electric charge (relative to ST) on the efficacy of treatments in RUL ECT appear to challenge our hypothesis, it is important to note that in most of them, average charge values did not exceed 504 mC, potentially avoiding the inhibitory range. Furthermore, electrode placement may modulate how charge affects therapeutic response. In both placements, the electric field of energy beyond ST covers the entire brain with an amplitude > 0.8 mA [23]. However, neuroimaging studies suggest that BL ECT induces bilateral hippocampal volume increases—a known predictor of clinical response—whereas RUL ECT tends to produce right-sided changes only [24,25]. These anatomical and neuroplastic differences may partly explain the divergent effects of high-dose stimulation across electrode placements [6,21].

The phenomenon observed in our study may also be explained by the different average charge used in each of the study groups. In the HCP group, the charge doses were consistently raised with each subsequent treatment, which resulted in an average value of 588.6 mC per session. On the other hand, in the DTP group, the charge dosage directly resulted from individual ST, and was 2.5 times higher, with an average of 196.6 mC per session. In general, the charge dose is calculated by the multiplication of a constant amplitude of 0.9 mA by the pulse width, twice the frequency, and the duration of the stimulation. Each of the aforementioned factors has a significant impact on the excitability of neurons and can consequently influence the occurrence or efficacy of the epileptic seizures [23]. ECT devices mainly work by increasing the number of pulses as the dose of the applied charge is increased, up to the limit in which the stimulus duration reaches 8 s. Subsequently, there is an increase in the frequency of pulses. With the use of high charges (especially more than 504 mC), the frequency of delivered pulses increases. It was reported that the frequencies exceeding 50 Hz suppress the ongoing seizure activity during ECT [26]. We hypothesize that once the ST is reached during ECT, additional stimulation—potentially delivered at higher pulse frequencies—may exert an inhibitory effect on ongoing ictal activity. It is possible that, after the ST is reached, further stimulation with high frequency can cause imbalance between the excitatory glutamatergic neurons and inhibitory GABAergic neurons [27]. Speculatively, seizure-inhibitory processes may scale with the time spent stimulating after crossing the ST. If so, administering charges > 504 mC could reflect parameter combinations (e.g., higher pulse frequency and/or longer train duration) that preferentially recruit inhibitory mechanisms and attenuate ictal expression, potentially reducing ECT efficacy.

Indeed, in our study, the HCP group had significantly lower remission rates than the DTP group, supporting this hypothesis. Nevertheless, due to the retrospective design of our study, we are unable to fully investigate this mechanism. Further clinical trials are warranted—for example, a randomized controlled trial assessing EEG seizure parameters with the same ST multiplication factor but different preset frequencies across groups.

### 4.1. Clinical Implications

Our findings suggest that using protocols leading to high absolute charges in BL ECT can be detrimental not only for seizure duration but also for the overall effectiveness of the treatment. In fact, using charges well-above the ST is associated with administering high-frequency pulses in long duration trains, which in our opinion is responsible for the worsening of the overall ECT performance. We suggest that in the BL ECT, the dosing of charge should be closely related to individual ST, and charges higher than 504 mC should be used only in patients with an extremely high ST. Nevertheless, further randomized controlled trials should explore the relation between charge, frequency of pulses, train duration after reaching the ST, and effectiveness of BL ECT.

### 4.2. Strengths and Limitations

The main strength of our study is the unique design that allows us to evaluate the use of very high charges on the effectiveness of BL ECT compared to commonly used dose-targeting algorithms based on individual ST. To our best knowledge, this is the first study in BL ECT with a constant increase in charge dosage protocol in which administering high charges is shown to have an impact on the effectiveness of ECT. However, there are several limitations to our study. Firstly, this is a study of retrospective design, which is inevitably associated with a greater risk of bias than randomized clinical trials. One of the more common sources of bias is selection bias. To avoid this, all patients who had undergone BL ECT and had a diagnosis of depression were included in our study. To minimize the risk of confounding factors, we performed a series of sensitivity analyses, in which no impact on the described relationships was detected. In the additional logistic regression analysis, only the DTP was a significant predictor of the outcome, which aligns with our hypothesis that a high charge can be detrimental for ECT effectiveness. Nevertheless, given the wide CI of the OR for the type of protocol and low number of participants, our findings should be interpreted with caution.

Secondly, due to its retrospective design, we were unable to perform systematic cognitive assessments. Memory impairment, particularly in BL ECT and with higher cumulative doses, is a well-known adverse effect that was not evaluated here. This represents a clinically relevant limitation, especially considering recent guidelines, which recommend routine objective and subjective memory monitoring throughout ECT treatment courses [28]. Our dataset did not include pulse frequency or train duration, and among stimulation parameters, only total charge and pulse width were available. Accordingly, we were unable to directly assess the hypothesis that higher pulse frequencies may modulate treatment effectiveness or the duration of seizures. In both groups, brief pulse ECT was used, but in the HCP group, pulse width varied from 0.5 to 1.0 ms due to the Double Dose program properties. The study by Arriba-Arnau et al. (2021) suggests that pulse widths of 1.0 ms and 0.5 ms in BL ECT provide comparable antidepressant efficacy [29]. However, the data also indicate the tendency for a higher ST with a 1.0 ms pulse width, which aligns with previous findings [23]. In our study, to counterbalance the potential increase in ST caused by the increase in pulse width, we used a different anesthetic regimen in the DTP group. Since no influence of etomidate or thiopental on the trajectory of depressive symptoms has been proven in the literature, and the data on their impact on seizure duration are inconclusive, we consider this a minor limitation [30,31,32,33]. Unequal distribution of age between the two groups could have a potential impact on antidepressant effectiveness or EEG parameters. This issue was addressed by adjusting the linear model to confounding factors such as age, history of ECT, total number of sessions, and interaction between the age and total number of sessions. The additional analysis suggests that the age difference between the groups did not have a significant impact on the results; however, given the abovementioned limitations of the study, these results should be interpreted with caution and require further confirmation in larger clinical trials.

Finally, assessment of the clinical response was based on retrospectively determined CGI-S and CGI-I scores, for which a single reviewer had to rely on the medical records. Therefore, inter-individual, subjective differences in clinical evaluation of patients’ conditions by clinicians cannot be ignored and could have influenced the ratings. Nevertheless, this method of retrospective assessment of patient records to evaluate the CGI scores has been used in research before. Due to the lack of reliable data on speed of response or cognitive side-effects, we did not assess the impact of high-charge dosage on those.

### 4.3. Conclusions

The findings from our study suggest that in BL ECT, using the protocol based on administering high charges, often exceeding 504 mC, may have a detrimental impact on both the ECT effectiveness (lower remission rate) and EEG parameters (shorter seizure length). Based on our findings, we suggest adjusting dosing in BL ECT to individual ST, and to avoid exceeding 504 mC as the average charge in the whole course of treatment. Because our findings are based on a retrospective analysis of data, there is a need for further prospective studies to optimize dosing protocols in BL ECT.

## Figures and Tables

**Table 1:** Baseline characteristics of depressed patients treated with bilateral ECT.

	High-Charge Protocol [n = 53]	Dose Titration Protocol [n = 22]	*p*-Value
Female [%; n]	62.3% [n = 33]	72.7% [n = 16]	*p* = 0.28
Mean age in years, [mean ± SD]	54 ± 15	41 ± 17	*p* = 0.001
Diagnosis of MDD * [%; n]	50.9% [n = 27]	50% [n = 11]	*p* = 0.57
Index episode in weeks	4.5 [2–11.8]	8 [3–24]	*p* = 0.27
Duration of disease in years [Median; IQR]	15 [7–20]	9 [3–18]	*p* = 0.04
Psychotic features [%; n]	26.4% [n = 14]	45.5% [n = 10]	*p* = 0.09
Comorbid substance use disorder [%; n]	9.4% [n = 5]	13.6% [n = 3]	*p* = 0.59
Comorbid anxiety disorder [%; n]	7.5% [n = 4]	13.6% [n = 3]	*p* = 0.41
Comorbid personality disorder [%; n]	13.2% [n = 7]	13.6% [n = 3]	*p* = 0.96
History of previous ECT [%; n]	15.1% [n = 8]	13.6% [n = 3]	*p* = 0.87
CGI-S—baseline [Median; IQR]	5 [5–6]	6 [5–6]	*p* = 0.003

CGI-S—Clinical Global Impression-Severity Scale; ECT—electroconvulsive therapy; MDD—major depressive disorder; * other patients were diagnosed with bipolar depression.

**Table 2:** Clinical outcome of ECT treatment with High-Charge Protocol and Dose Titration Protocol.

Groups	High-Charge Protocol (n = 53)	Dose Titration Protocol (n = 22)	*p*-Value	Adjusted *p*-Value
CGI-S—endpoint [Median; IQR]	3 [2–3]	2 [1.8–2.3]	*p* = 0.003	*p* = 0.02 *
CGI-I [Median; IQR]	2 [1–2]	1 [1–2]	*p* = 0.007	*p* = 0.004 *
Remission rate [%]	43.4% (n = 23)	77.3% (n = 17)	*p* = 0.007	-

CGI-S—Clinical Global Impression-Severity Scale; ∆CGI-S—change in Clinical Global Impression-Severity Scale; CGI-I—Clinical Global Impression-Improvement Scale. * Adjusted for age, CGI baseline, length of disease, psychotic features, history of ECT, total number of sessions, comorbid anxiety, addiction, personality disorder.

**Table 3:** Multivariable logistic regression model predicting remission status selected in backward likelihood-ratio modeling.

	B	OR	95% CI	*p*-Value
Dose Titration Protocol	1.76	5.81	1.48–22.86	*p* = 0.01
Comorbid Anxiety Disorder	−1.78	0.17	0.02–1.17	*p* = 0.07

Cox and Snell R Square = 0.14; Chi Square = 10.14; df = 2; *p* = 0.006.

**Table 4:** Characteristics of ECT parameters in High-Charge Protocol and Dose Titration Protocol groups.

	High-Charge Protocol (n = 53)	Dose Titration Protocol (n = 22)	*p*-Value	Adjusted *p*-Value
Total Charge over course (mC) [Median; IQR]	5663.1 [3894.6–7492.7]	2088.4 [1375.3–2914.7]	*p* < 0.001	*p* = 0.005 *
Total number of sessions	10.3 ± 2.3	11 ± 2.3	*p* = 0.23	*p* = 0.98 *
Average charge/session (mC) [mean ± SD]	555.7 ± 147.1	208.5 ± 88.9	*p* < 0.001	*p* < 0.001 *
Total EEG seizure length (s) [Median; IQR]	399 [300–480.5]	675.5 [547.5–943.5]	*p* < 0.001	*p* < 0.001 *
EEG seizure length/session (s) [Median; IQR]	38.4 [33.8–47.1]	68.6 [52.7–84.7]	*p* < 0.001	*p* < 0.001 *
Total PSI (%) [Median; IQR]	460.3 [255–523.1]	457.6 [322.8–606.7]	*p* = 0.94	*p* = 0.72 *
PSI/session (%) [Median; IQR]	74.2 [65.8–82.0]	63.6 [55.8–74.4]	*p* = 0.02	*p* = 0.003 *
MIA (uV) [Median; IQR]	1699.5 [1445.9–2200.5]	2108.9 [1681.3–2495.5]	*p* = 0.02	*p* = 0.98 *
MIA/session(uV) [Median; IQR]	183 [158.1–216.4]	208.8 [171.9–239.3]	*p* = 0.06	*p* = 0.84 *

EEG—electroencephalography; MIA—midictal amplitude; PSI—postictal suppression index. * Adjusted for age, CGI baseline, length of disease, psychotic features, history of ECT, comorbid anxiety, addiction, personality disorder after Benjamini–Hochberg correction for multiple testing.

## Data Availability

The original contributions presented in this study are included in the article/supplementary material. Further inquiries can be directed to the corresponding author(s).

## Supplementary Materials

The following supporting information can be downloaded at: https://www.mdpi.com/article/10.3390/jcm14186490/s1, Table S1: Initial logistic regression model; Table S2. Summary of studies on the impact of multiplication of seizure threshold on effectiveness of BL ECT.
