# Supplementary material for: Comparison of High-Charge Protocol vs. Dose Titration Protocol in Bilateral ECT: Evaluation of Antidepressant Effectiveness and EEG Parameters
Source: J Clin Med. 2025 Sep 15;14(18):6490. doi: 10.3390/jcm14186490 (PMC12470770; doi:10.3390/jcm14186490)
Supplement: Supplementary file 1 [file jcm-14-06490-s001.zip › jcm-3744171-supplementary.pdf]

**Supplementary Table S1. Initial logistic regression model.**

| Predictor | B | OR | 95% CI | p-value |
|---|---|---|---|---|
| **Dose titration method** | 2.03 | 7.47 | 1.39- 41.91 | **p=0.02** |
| **Age** | 0.007 | 1.08 | 0.96-1.06 | p=0.77 |
| **Length of disease** | -0.03 | 0.97 | 0.91-1.06 | p=0.37 |
| **Psychotic features** | -0.004 | 0.99 | 0.21-4.69 | p=0.99 |
| **CGI-S baseline** | -0.13 | 0.88 | 0.34-2.31 | p=0.79 |
| **Comorbid anxiety disorder** | -1.87 | 0.16 | 0.02-1.4 | p=0.1 |
| **Comorbid substance use disorder** | -1.41 | 0.24 | 0.03-1.83 | p=0.17 |
| **Comorbid personality disorder** | -0.781 | 0.458 | 0.072-2.914 | p=0.41 |
| **History of previous ECT** | 0.267 | 1.307 | 0.278-6.138 | p=0.73 |

Cox & Snell R Square= 0.18; Chi Square = 13.681; df=9; p=0.13. CGI-S – Clinical Global Impression Scale Severity, ECT – Electroconvulsive therapy.

**Supplementary Table S2. Summary of studies on the impact of multiplication of seizure threshold on effectiveness of BL ECT.**

| Author and year of publication | Study design | Electrode placement | Study cohort (n; diagnosis; age±SD) | Dosing strategy | Average charge (mC, ±SD) | Average seizure length (s, ±SD) | Outcome |
|---|---|---|---|---|---|---|---|
| **Ju et al. (2005)** | Retrospective analysis of two groups: LCG<504 mC and HCG>504 mC | BL | 59 patients with MDD; mean age 60±13.2 years. | ST measured on first session; Treatment dose: 1.5xST | LCG: 201±104.2; HCG: 705.6±138.6 | N/D | Response rate to ECT did not significantly differ between LCG and HCG (67% vs.85%). Remission rate did not significantly differ between LCG and HCG (37% vs 46%). |
| **Ottosson et al. (1960)** | RCT; 3 groups (A, B, C) randomly allocated to receive different ECT protocols | BL | 69 patients with MDD; mean age: Group A: 54.4±10.9; Group B: 57.1±8.7; Group C: 52.2±9.3 years. | Age-based ST (stimulus time in s equal to age in decades). Treatment dose: Group A: ST; Group B: 1.25-1.5xST; Group C: 1.25-1.5xST+lidocaine | N/D | Total duration of seizure: Group A: 365±165 s; Group B: 366 ± 168s; Group C: 226 ± 107s. | After completion of the treatment series, improvements in depression scores and total scores did not differ significantly among groups A, B, and C. The total number of treatments was significantly higher in group C than in group A and B. In comparison with groups A and B, the total duration of seizure discharges was significantly shorter in group C both after four treatments and, despite the higher number of treatments, after the completed series. |
| **Chanpattana et al. (2000)** | RCT; ECT + flupenthixol; 3 groups randomly allocated to receive different ECT protocols | BL | 62 patients with schizophrenia; mean age: Group 1 ST: 35.1±8.3; Group 2 ST: 35.2 ±8.2; Group 4 ST: 33.5 ±7.4 years. | ST measured on first two session Treatment dose: Group1xST, Group 2xST, Group 4xST | Group 1xST: sessions 2-9: 118.4±47.1, sessions 11-end: 254.6±131. Group 2xST: sessions 2-9: 195.6±32.9, sessions 11-end: 413.1±139. Group 4xST: sessions 2-9: 372.2±66.5, sessions 11-end: 576 (all patients). | N/D | Equal remission rates among the three treatment groups 1xST, 2xST, and 4 xST. Remitters in both the 2xST and 4xST groups received fewer ECT treatments at the time of first significant clinical improvement, defined as a BPRS score of 25 or less. Accordingly, at the end of the study, remitters in both the 2xST and 4xST groups received fewer ECT treatments. |
| **Krystal et al. (2000)** | Retrospective analysis | RUL and BL | 388 patients with MDD or BD; mean age: HCG: 63.9±16.2; LCG: 57.6±18.1 years. | ST measured on first session; treatment dose: RUL: 2.25xST; BL: 1.5xST | 72 patients received charge of 572 | 22 patients ictal activity <25 s | Reduced response in patients needing supra maximal stimulus intensity (32% vs. 66%). |
| **Brus et al. (2017)** | Retrospective analysis | RUL, BL and BF (90% RUL) | 1671 patients with MDD or BD; mean age: 53.9±17.6 years. | N/D | BL: 409±168; RUL: 335±145; BF: 278+84. | N/D | Patients with psychotic features and older age were predictive of higher remission rates after ECT. Pulse width >0.5 and higher charges were associated with higher remission rates. |
| **Sackeim et al. (1993)** | RCT; 4 groups randomly allocated to receive different ECT protocols | RUL and BL | 96 patients with MDD; mean age: RUL-LCG: 57±16; RUL-HCG: 56±15; BL-LCG: 60±13; BL-HCG: 53±15 years. | ST measured on first session; treatment dose: RUL-LCG: 1xST; RUL-HCG: 2.5xST; BL-LCG: 1xST; BL-HCG: 2.5xST | RUL-LCG: 86±34; RUL-HCG:175±75; BL-LCG: 212±176; BL-HCG: 321±155. | RUL-LCG: 57±19; RUL-HCG: 60±20; BL-LCG: 57±17; BL-HCG: 58±16. | Immediately after ECT, response rate did not significantly differ between RUL-HCG (70%), BL-LCG (70%) and BL-HCG (70%). Only RUL-LCG had significantly lower response rates (22%), but also lower cognitive side effects. Absolute electrical dose was not related with the outcome, rather, the key factor was whether the dosage substantially exceeded the ST. |

BD - Bipolar disorder; BF - Bifrontal ECT; BL - Bilateral ECT; ECT - Electroconvulsive therapy; mC - Milicoulombs; HCG - High charge group, LCG - Low charge group; MDD - Major depressive disorder; N/D - no data; RCT – Randomized controlled trial; RUL - Right unilateral ECT; ST - Seizure threshold.
